# Rapid Numerical Estimation of Pressure Drop in Hot Runner System

**DOI:** 10.3390/mi12020207

**Published:** 2021-02-18

**Authors:** Jae Sung Jung, Sun Kyoung Kim

**Affiliations:** Department of Mechanical System Design Engineering, Seoul National University of Science and Technology, Seoul 01811, Korea; sungs@seoultech.ac.kr

**Keywords:** injection molding, OpenFOAM, pressure drop, cross model, generalized Newtonian fluid, hot runner

## Abstract

To determine dimensions in the hot runner systems, given a material, it is necessary to predict the pressure drop according to them. Although modern injection molding simulators are able to evaluate such pressure drops, they are expensive and demanding to be employed as a design utility. This work develops a computer tool that can calculate a pressure drop from the sprue to the gate assuming a steady flow of a generalized Newtonian fluid. For a four drop hot runner system, the accuracy has been verified by comparing the obtained results with those by a commercial simulator. This paper presents how to utilize the proposed method in the hot runner design process.

## 1. Introduction

A hot runner system (HRS) is widely adopted in modern injection molding processes. The HRS allows higher productivity, easier quality control, and apparent resin cost saving. Rapid product cycle demands the lead time be shorter and shorter while the reliability of mold tooling is always important. As a result, the design process should be quick as well as systematic. In an HRS design, there are two important functions that should be guaranteed. The first one is to maintain the target temperature within a tolerable range. Second, the runner system has to deliver melt in an efficient as well as balanced fashion. This work is focused on the second part. The dimensions of the manifold and the nozzles should be optimally determined considering both the allowable pressure drop and the necessary flow rate [1].

The pressure drop is especially important in microsystem. Injection molding of a micro-device requires a high pressure at the gate to drive the filling flow in the micro-cavity [2,3,4,5]. To maintain the required pressure at the gate, the pressure drop through the runner should be suppressed. The diameter of the runner should be increased while the length should be shortened. However, the length is very difficult to shorten since it is supposed to set by the delivery requirement. In the meantime, the increase of the diameter causes serious problems which negatively impacts the molding process. The increased diameter adds volume to the runner. The pressure drops linearly along with the diameter while the volume increases quadratically. The increased volume per se is the situation that should be avoided. The melt retained in the runner will degrade while the stagnant phases. Moreover, the temperature can be non-uniform and fluctuating for wider runners. Thus, the runner diameter should not be bigger than required.

Technologies and studies of numerical filling simulations for conventional injection molding process have matured [2,3,6,7,8,9]. In other processes, molding optimization is an important issue [10]. It has been widely used for design of molds and products as well as for troubleshooting in the processes [11,12,13,14,15,16]. There are several CAE (computer aided engineering) packages, such as MoldFlow, Modex3D, and 3D Timon, which are commercially successful in industry and also widely accepted as research tools [2,9,17,18]. Especially, CAE has been utilized for cooling line design of injection molds [15,19]. It is also proven that it can handle fairly complicated HRs [20,21].

In an HR design, it is desirable to maintain sufficient flow conductance with limited pressure [1]. The pressure drop is always an important matter of concern in HR both in the sequential and conventional gating methods [20,22,23]. Given a flow rate, the pressure drop can be obtained by a numerical simulation, which can be conducted in many commercial CAE programs.

However, there are several difficulties in calculating the pressure drops for design purpose using such programs. First of all, it is necessary to train the design engineers to let them learn the entire CAE process from three-dimensional drawing to simulation. Second, whenever the dimensional parameters are changed, the mesh should be regenerated repeatedly. It is thought that the HR design engineers would not be willing to repeat the meshing. Rather, they would reuse existing design dimensions or change the dimensions by a rule of thumb without a scientific analysis. Third, the simulation itself takes a quite long time for each case. Even when the mesh is ready, one simulation run takes quite a long time to be used while designing a hot runner. Fourth, such simulation programs are expensive to purchase and costly to operate. To allow all the in-house design engineers to access the simulation program, multiple licenses should be purchased. This is impracticable to most HR providers.

As a result, the pressure drop is suggested to be calculated based on analytical methods in the design phase [24,25]. The analytical approach cannot consider the pressure drops due to directional changes and flow distributions. Moreover, the viscosity approximation, which is conducted by the power law or other shear thinning models, can induce additional errors. Once the dimensions are set, a runner designer can move onto numerical simulation for verification. However, this can cause laborious trial and errors.

Thus, a computer tool dedicated to flow design of HRS would help the design process. However, such a tool can hardly be found in the literature. To expedite the design process of HRS, this work proposes a rapid numerical method for calculating the pressure drop while melt flows through the hot runner. This work focuses on calculation of the melt flow in HR for HR field designers. The whole flow field will be sectioned into several subdomains, and then the pressure drop will be calculated for each subdomain. Afterwards, the total pressure drop can be estimated by summing up the pressure drops in the subdomains. Given the melt flow rate and geometric setup, the proposed system will yield the pressure drop in affordable time. The method will be implemented using a spread sheet computer software and a publicly available CFD (computational fluid dynamics) computer program. A CFD computer program usually numerically solves the Navier–Stokes problem by the finite volume (FV) or finite element (FE) methods. A public FV-based CFD program, OpenFOAM, was employed to allow other engineers or researchers to easily reproduce our work. It is expected that this work would contribute to reduction of the HRS design time.

## 2. Numerical Methods

### 2.1. Overall Approach

The hot runners include two major parts, which are the manifold and nozzles. They have several typical forms especially depending on the number of nozzles. The HR providers have their own product lines, which are internally standardized and presented in their catalogues. Most of the orders fall into the standards. Therefore, a flow simulation tool that is dedicated to flow through the manifold and the nozzle can be developed taking the standard geometries into account. The basic approach here is to divide the entire flow path into a number of subdomains followed by assessing the pressure drop in each subdomain during a saturated flow of a generalized Newtonian fluid (GNF). The GNF models can well represent rheological behaviors of molten polymers that can be assumed as inelastic non-Newtonian fluids. Then, the whole pressure drop is estimated by adding up the pressure drop in all the subdomains. This kind of method has been widely exploited in many pipe network designs [26].

**Figure 1:**
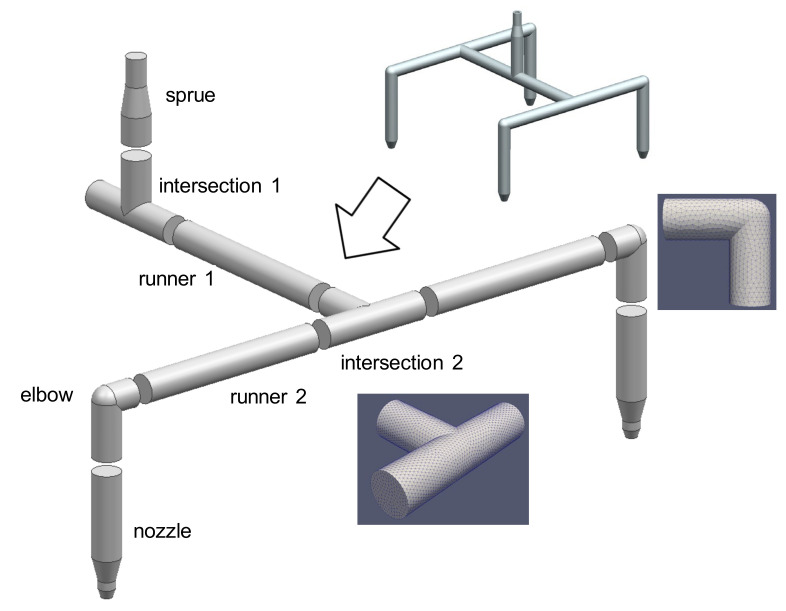
Division of a typical hot runner with four drops into seven subdomains.

Figure 1 shows a typical hot runner layout with four drops. There are four straight sections with a sprue section, two intersections, one elbow, and the nozzle. In most parts of the flow path, the cross-section is circular. For a fairly long circular pipe section, the pressure drop can be analytically calculated assuming fully developed flow of power-law fluid (PLF), which will be utilized here. In the proposed method, the pressure drop in each section is calculated separately and added up to assess the pressure drop throughout the whole runner. Several different kinds of HRS will be treated including the one shown in Figure 1.

Consider design parameters that determine the geometry shown in Figure 1. A set of design parameters are predefined as

(1) $$g=\left\{g_{1},g_{2},\ldots ,g_{N}\right\}$$

In a subdomain indexed *i*, the pressure drop will be $\Delta p_{i}\left(g\right)$ and the total pressure drop is represented as

(2) $$\Delta p_{total}=\sum_{i=1}^{N}\Delta p_{i}\left(g\right)$$

The purpose of this work is to calculate the total pressure drop, $\Delta p_{i}\left(g\right)$, under a given set of design parameters, **g**. An HR designer will be able to check the change in $\Delta p_{total}$ due to the change in any design variable, $g_{i}$.

### 2.2. Assumptions

**Figure 2:**
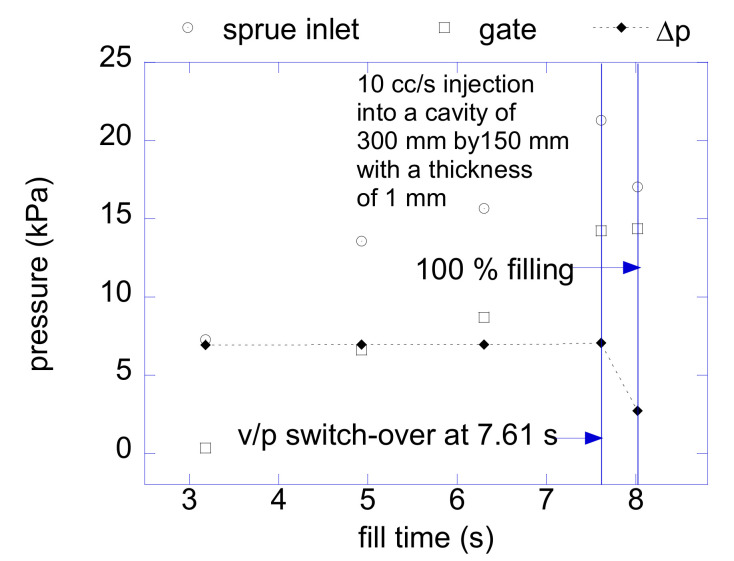
Pressure drop over the filling time at 100 cc/s injection.

This work argues that the total pressure loss for driving the HR flow can be assessed by solving a steady saturated flow of each subdomain followed by adding up the pressure drops. Let us first discuss the assumption of a saturated steady flow. The flow between the sprue to the gate in HR can be considered steady if the flow rate is constant since the downstream cavity flow normally cannot affect the upstream HR flow. In an injection molding process, the melt flow is inherently unsaturated and the melt front poses a moving boundary inside a mold cavity. A hot runner needs to be flow-conductive enough to reserve the pressure head for filling the cavity and for transmitting sufficient packing pressure during the post-filling phase. Regardless of the degree of filling, the pressure drop from the sprue to the gate does not significantly vary while injection rate is maintained constant. Figure 2 shows the pressure drop during filling of a sample cavity. It was obtained by Autodesk MoldFlow Insight 2012, which will be referred to as MoldFlow in the rest of this paper. The geometric model is shown in the inset of Figure 1. The pressure drop in HRS is maintained almost constant during filling until the switch-over. Thus, it is reasonable to estimate the pressure drop throughout a hot runner during mold filling based on a saturated steady flow model. As a design method for HRS, it will be a viable and effective method. Moreover, it should be noted that the pressure drop before the switch-over is likely to be the highest since that begins to decrease from the switch-over point.

**Figure 3:**
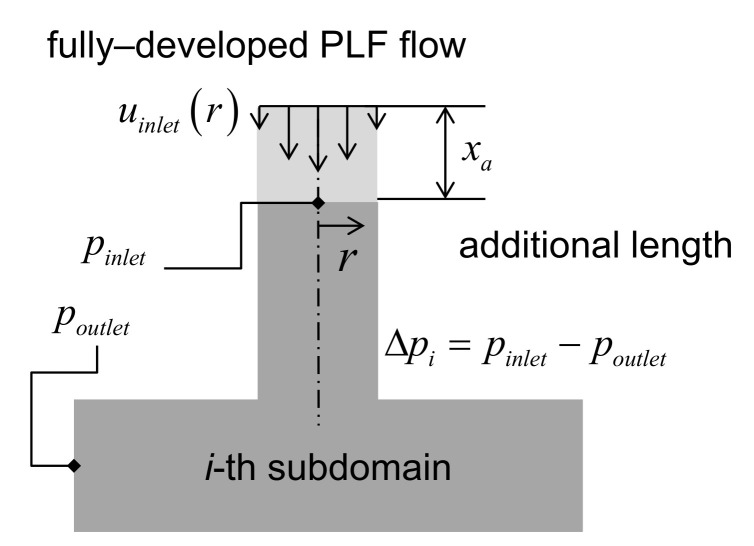
Scheme of pressure drop calculations.

The next matter is to justify division of the whole domain into several subdomains. The benefits from this include faster computation, facilitation of parallel computation, easier mesh handling, and two-dimensional approximation for axisymmetric subdomains. Here, two approximations are required. First, in each subdomain, a fully-developed velocity profile of the PLF will be imposed on the inlet boundary with some additional length as shown in Figure 3. In the first subdomain of the inlet, as long as the flow in the injection molding machine is not analyzed, a fully-developed profile is the best condition imposable here. In the middle subdomains, the velocity of the adjacent upstream outlet can be possibly imposed. However, that way is not chosen in this work since it requires sequential computation and prevents connection between axisymmetric and three-dimensional subdomains. As a result of this approximation, any secondary flows, which are perpendicular to the primary flow, cannot be relayed on the interface between the subdomains. Especially when one of the adjacent subdomains is curved, there should be a secondary flow and it is known to contribute to the pressure drop. However, the effect of the secondary flow on the interface will be negligible since most of the pressure drop due to the secondary flow will be taken into account in calculation within the curved sections and the interfaces will be far downstream from the curved section. Second, the absolute pressure cannot be accurately obtained by this method. Thus, dependency of the viscosity and the density on pressure cannot be considered. This is assumed in many Newtonian incompressible flows. However, in injection molding simulation, the pressure effects have been taken into consideration, although it is not significant in the filling phase. The validity of these assumptions will be checked out by comparing the pressure drops with those of fully three-dimensional analyses.

### 2.3. Governing Equations

A typical formalism for momentum transport of a generalized Newtonian fluid (GNF) is reproduced here. This work adopts an isothermal three-dimensional steady model. This work ignores viscous heating although it is important in HR to reduce the computational time, which will be discussed later. Consider the velocity vector **u**, pressure *p*, and density *ρ*. Neglecting body force, a steady-state momentum equation for a GNF is

(3) $$\rho u_{k}\frac{\partial u_{i}}{\partial x_{k}}=-\frac{\partial p}{\partial x_{j}}\delta_{ij}+\frac{\partial \tau_{ij}}{\partial x_{j}}$$

Here, for a given temperature *T*, the shear stress tensor $\tau_{ij}$ is expressed as

(4) $$\tau_{ij}=2\eta (\dot{\gamma },T)d_{ij}$$

where $d_{ij}$ is the rate of deformation tensor, which is of the form

(5) $$d_{ij}=\frac{1}{2}\left(\frac{\partial u_{i}}{\partial x_{j}}+\frac{\partial u_{j}}{\partial x_{i}}\right)$$

The shear rate, $\dot{\gamma }$, which is the second invariant of $d_{ij}$, is given by $\dot{\gamma }={\left(2d_{ij}d_{ji}\right)}^{1/2}$.

### 2.4. Viscosity Model

In this work, the power law model and the cross model are employed to represent the viscosity. The power law model is expressed as

(6) $$\eta (\dot{\gamma })=K{\dot{\gamma }}^{n-1}$$

where *n* and *K* are the power-law index and the consistency, respectively.

The most widely employed viscosity model for simulation of injection molding is the Cross-WLF model, which will more realistically represent the viscosity especially near the first Newtonian plateau. To utilize the existing viscosity data in that form, the hot runner pressure drop calculator (HRPDC) will also allow input of the Cross model, which takes the form of

(7) $$\eta =\frac{\eta_{0}}{1+{\left(\eta_{0}\dot{\gamma }/\tau \right)}^{1-n}}$$

where *τ* is a curve-fitted constant for a specific polymer and $\eta_{0}$ is the zero-shear viscosity. This study does not solve energy equation. However, the HR temperature significantly affects the pressure drop by changing the viscosity. Thus, it will be an important input value to the hot runner pressure drop calculator (HRPDC). Sometimes, the high pressure of the melt resident in the hot runner noticeably increases the viscosity. The WLF model is employed to represent $\eta_{0}$ as a function of pressure and temperature, which is of the form

(8) $$\eta_{0}\left(T,P\right)=D_{1}\exp\left[-A_{1}(T-D_{2})/(A_{2}+T-D_{2})\right]$$

Here, *D*_1_, *D*_2_, *A*_1_, and *A*_2_ are constant values that should be determined from experimental measurements for a specific polymer.

In the circular straight section, an analytical solution is available in a simple closed form for PLF but not for CLF (Cross law fluid). Note that the aforementioned CFD computer program, OpenFOAM, will solve Equation (3) with Equation (7).

### 2.5. Boundary Conditions

The boundary conditions are quite simple since there is no free boundary in this model. The boundary conditions are the same for all subdomains. First, a no-slip condition is imposed on the walls.

(9) u=0 on the walls

Second, a Neumann condition is imposed on the outlet.

(10) $$\frac{\partial u}{\partial n}=0\;\mathrm{on}\;\mathrm{the}\;\mathrm{outlet}$$

where *n* is the coordinate variable normal to the wall along the normal vector **n**. Moreover, a reference cell for pressure field is chosen on this boundary and a gauge pressure of zero is imposed. Thus, $p_{outlet}$ in Figure 3 is set as zero. As a result, the pressure at the inlet will be the pressure difference. Third, a velocity profile should be imposed in the inlet. In every subdomain, the flow starts again with an inlet velocity.

(11) $$u_{n}=u_{inlet}\left(r\right)\;\mathrm{on}\;\mathrm{the}\;\mathrm{inlet}$$

The easiest way is to simply impose a uniform velocity throughout the boundary.

$$u_{inlet}\left(r\right)=u_{mean}$$

where the uniform velocity is of the form

(12) $$u_{mean}=\frac{4Q}{\pi D^{2}}$$

where *Q* and *D* are the flow rate and the diameter, respectively.

The velocity field between the subdomains should be fully developed. Thus, it is necessary to add more length to the beginning part of each subdomain. For a laminar flow, the entry length is roughly

(13) xfd≈0.05DReD

The calculated value of $x_{fd}$ cannot be larger than *D* in HR melt flow of any thermoplastics however fast the melt flow is. Hence, the additional length, ahead of the actual interval for pressure calculation is set as

(14) $$x_{a}\;=\;D$$

Although this would probably be enough, to guarantee the fully developed flow at the point of the inlet pressure measurement, a fully developed velocity profile of a PLF is imposed instead of a flat profile of $u_{mean}$, which is

(15) $$u_{inlet}\left(r\right)=u_{mean}\frac{1+3n}{1+n}\left[1-{\left(\frac{r}{R}\right)}^{\frac{n+1}{n}}\right]$$

where *R* is the radius of the runner.

By doing so, for a PLF, $x_{a}$ can be set equal to 0, and for a CLF, the velocity will rapidly develop to a fully developed profile of a CLF while flowing the additional length. It is assumed that any velocity components other than normal to the inlet surface do not significantly contribute to the pressure drop near the connecting boundary, which will be examined in test cases. Moreover, given a flow rate and length, the pressure drop in a linear runner flowing PLF is obtained simply as

(16) $$\Delta p=\frac{2\eta_{0}L}{R}{\left[Q\frac{1+3n}{n\pi R^{3}}\right]}^{n}$$

## 3. Implementations

### 3.1. Work Flow

To calculate the pressure drop according to design parameters, there are four essential parts to be realized. First of all, the design variables are required to be set to determine the geometry of the HR in calculation. Second, the computational mesh should be accordingly built followed by imposition of the boundary conditions. This should be automatically done without any manual intervention of operator. Third, the pressure field of melt flow should be solved in a fastest possible way. Fourth, the calculated pressure at the desired locations needs to be retrieved. They should be conducted in a sequential manner as shown in Figure 3. To facilitate these four steps, a spreadsheet program for PC, Microsoft Excel 14.0, and a CFD (computational fluid dynamics) computer program, OpenFlow 5.3, were employed. It is a Microsoft Windows version of OpenFOAM 2.1 ported and compiled by Symscape [27]. In the following, it will be referred to as OpenFOAM since this name is widely known in the CAE industry. The VBA (visual basic for applications) scripting in Excel and components in OpenFOAM will be utilized for presentation, interfacing, communication, meshing, and calculation.

### 3.2. Overall Architecure

**Figure 4:**
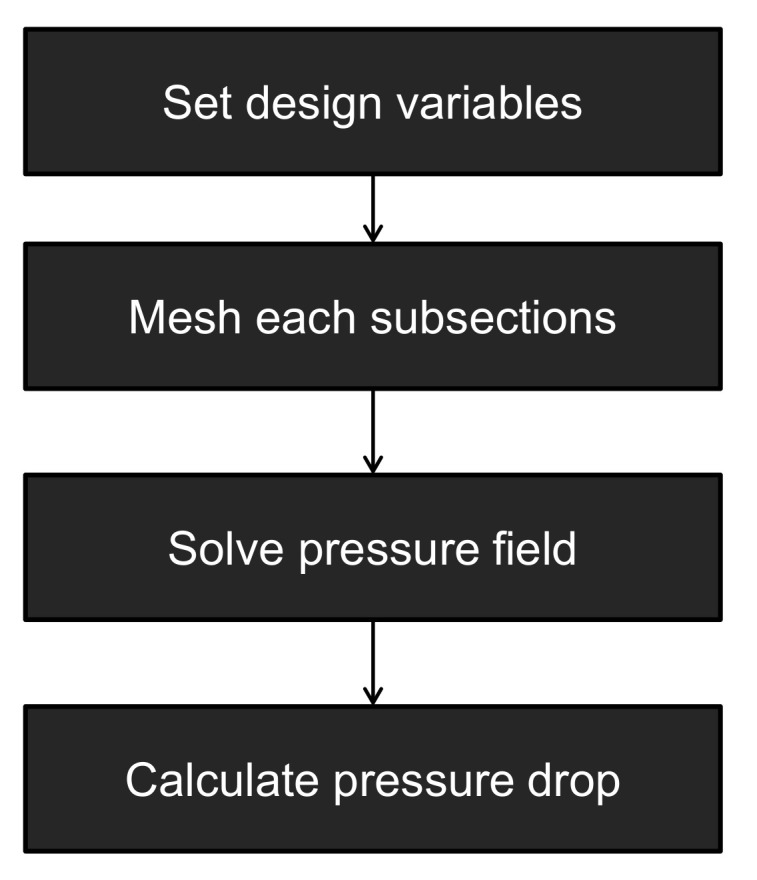
Procedures of pressure drop calculation.

The spreadsheet is the control center of the HRPDC, which receives user input and order, executes OpenFOAM components, collects the calculated pressure by reading the OpenFOAM output file, and presents the results. In actual spread sheet pages, the material properties and the geometric information are specified in the designated cells. Figure 4 shows the architecture of HRPDC in an implementational level. This shapes up the work flow shown in Figure 3 realizing executions and communications required for each procedure. Excel and OpenFOAM communicate with one another by the following two ways. Since OpenFOAM is comprised of files executable in the command line, a VBA function in Excel that enables running a command line executable is utilized when Excel has to call a component of OpenFOAM. Given that no interface allows OpenFOAM to access Excel, Excel has to read the files written by OpenFOAM components after the execution. This file level communication is primitive in terms of software engineering but works smoothly and reliably.

**Figure 5:**
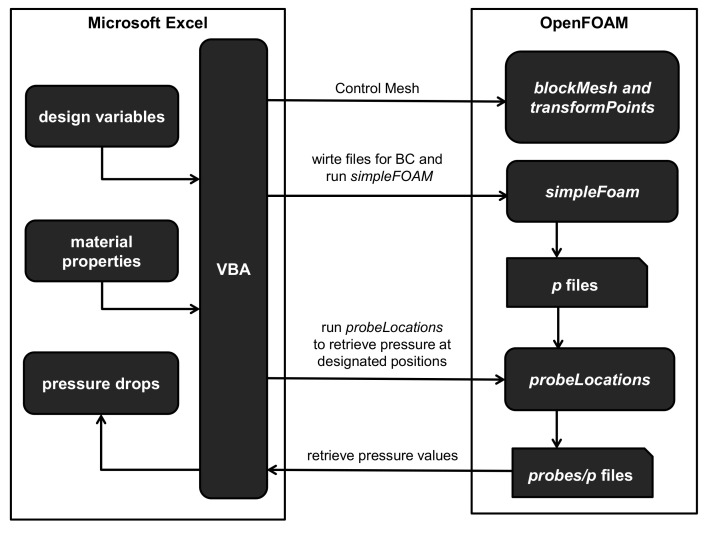
Schematic diagram for HRPDC.

The structure of HRPDC has been schematically shown in Figure 5. A user is required to set the design variables and the material properties in advance to any action. When the user is done with the input and ready for calculating the pressure, the user needs to initiate calculation by pressing a button, which actually starts a VBA code. The VBA code should also write the input files for OpenFOAM. Based on the design variables, the VBA code creates a new mesh or deforms an existing mesh. Moreover, it also writes boundary conditions and material properties as OpenFOAM requires. On completion of the calculation, the pressure drops need to be collected and summed up. In addition to communications, VBA also controls the sequence of procedures and performs minor computations. To retrieve the pressure value at the inlet and outlet, an OpenFOAM utility, probeLocations is employed. It writes interpolated pressure value at points designated by coordinate values. Then, Excel presents the pressure drop in the sheet.

### 3.3. Flow Calculation

This work employs the FVM (finite volume method) for solving the momentum equation, which accurately meets conservation of mass and momentum. It is well-established and nothing new here. The SIMPLE (semi-implicit method for pressure linked equations) method is employed to solve the prescribed momentum equation, which is a Navier–Stokes equation of shear-thinning liquid. In SIMPLE, a Poisson equation is repeatedly solved for the correction of pressure, which is inherently a parameter satisfying the continuity in a steady Navier–Stokes equation. The correction is fulfilled with an under-relaxation by adjusting the pressure to attain the divergence free state of the velocity field.

Regarding the numerical method itself, it is fully-fledged and widely available [28]. There are several open source codes that realize the SIMPLE or compatibles for similar flows. To sum up the all the pressure drops in the subdomains, the actual calculation is initiated and controlled in spreadsheet software. To do so, the calculation code should be callable from the spreadsheet. Moreover, to cope with many different meshes, it should be able to systematically handle meshing and afterwards modification without great user labor. An open source code CFD (computational fluid dynamics) toolbox, OpenFOAM (open source field operation and manipulation) provides all such capabilities. The simpleFoam code in OpenFOAM, has been utilized to solve the described problem. Its further details can be found in [27].

Since it solves an incompressible isothermal flow, it cannot accommodate the density change due to the high pressure during injection molding. From the computational aspect, if the compressibility is considered, it would not be a fast solver. Maintaining the computational efficiency, to take the density change into account, the current model will calculate the flow twice to correct the density change. In the initial run, it will solve the flow with the approximated density using Equation (16) and the following *pvT* equation.

(17) $$v(T,p)=v_{o}(T)\left[1-C\ln(1+\frac{p}{B(T)})\right]$$

where $v_{o}(T)=$$b_{1}+b_{2}\bar{T}$, B(T)=$b_{3}\exp\left[-b_{4}\bar{T}\right]$, and $\bar{T}=T-b_{5}$. Here, the constants, *b_i_*’s are fitted from the test [25,29].

Then, in the second run, the densities in each section are corrected again with the pressures from the initial run. For a slow flow rate, the density correction is not necessary since the density does not affect the flow due to negligible inertial effects. However, for a higher flow rate, the Reynolds number can be over 1 and the inertia term plays an ineligible role. In this case, density affects the calculated pressures values.

### 3.4. Properties

The melt properties such as viscosity and density are to be put into the sheets directly. For some representative thermoplastics, the properties are prewritten in the spreadsheet and can be selected in a dropdown box. This can be implemented simply by adding several procedures in the spreadsheet. For tests cases, a generic PP (polypropylene) from the MoldFlow database is employed [29]. The viscosity in a Cross-WLF(Williams-Landel-Ferry) form is presented in Table 1. Moreover, the *pvT* constants at the melt state for Equation (17) are shown in Table 2.

### 3.5. Geometries and Meshes

In contrast to properties handling, the geometries are fairly complicated to deal with. However, it is unnecessary to write plumbing codes for that since OpenFOAM provides several utilities that can manipulate input and output files. There are several subdomains that require three-dimensional meshing. Most three-dimensional meshes have to be built in advance while axisymmetric meshes can be created on demand. In most cases, such prebuilt three-dimensional meshes can be reused for different values of design variables simply by magnifying the entire mesh with the use of transformPoints utility in OpenFOAM. When the dimensions are set, the command is written in the batch file by VBA according to the dimensions to be executed under Windows CMD. The batch files are also called by VBA when the button in the Excel sheet is pressed. For axisymmetric sections, the mesh can be built in the runtime with negligible computational costs using the mesh tool in OpenFOAM, blockMesh. The VBA code also creates the blockMeshDict files according to the geometry. Then, the blockMesh utility is also written in the Windows batch file and then called by VBA. The axisymmetric domains are implemented using wedge patch [27].

**Figure 6:**
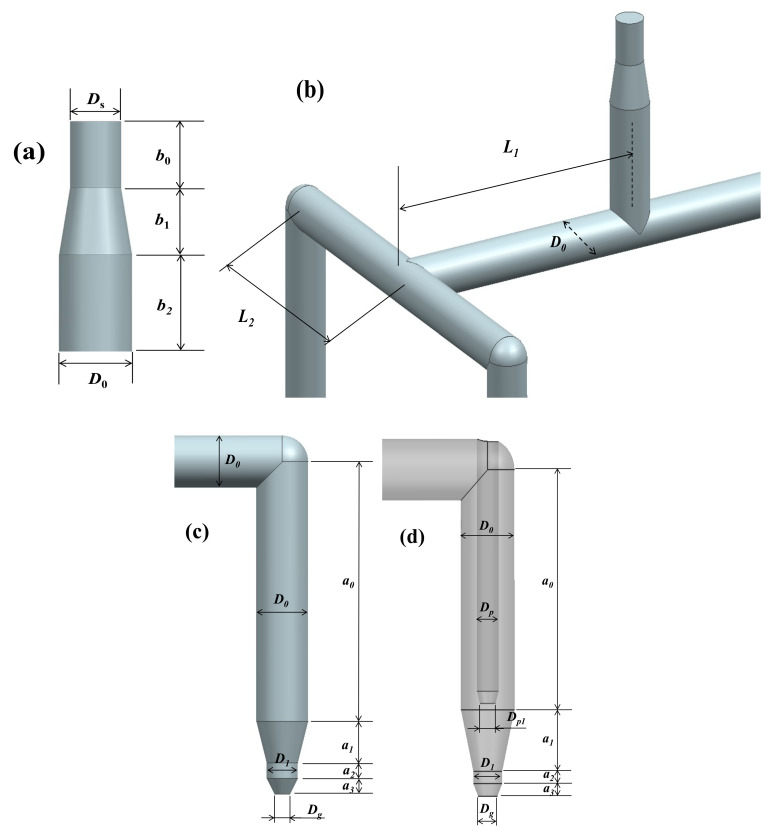
Major design variables: (**a**) sprue; (**b**) runners; (**c**) nozzle without valve; and (**d**) nozzle with valve.

Consider the HR shown in Figure 1. The geometric information of each subdomain is presented in Table 3. Note that among those four identical drops, only one is calculated because of symmetry. This case assumes the melt flow is equally distributed at every branch. Of course, a case with unbalanced HRS can be treated in the HRPDC. Figure 6 presents all the design variables for this HRS. Again, the purpose of the HRPDC is to calculate the total pressure drop on change of these variables.

Inevitably for a three-dimensional subdomain, a prebuilt mesh is required. Such subdomains are specified in Table 3. When a prebuilt mesh is magnified, both the length and diameter are enlarged at the same ratio. Consider the runner 1 between intersection 1 and intersection 2 in Figure 1. When $D_{0}$ is increased to $D_{0}^{\prime }$ with fixed *L*_1_ in Figure 6b, the length of runner 1, *L*_1*a*_, needs to be accordingly shortened. The length to be reduced is $4\left(D_{0}^{\prime }-D_{0}\right)$ or the final length is $L_{1}-4D_{0}^{\prime }$. Since the mesh of the runner 1 can be easily built in the runtime, such change in length is not a problem.

## 4. Results and Discussions

### 4.1. Simple Verifications

**Figure 7:**
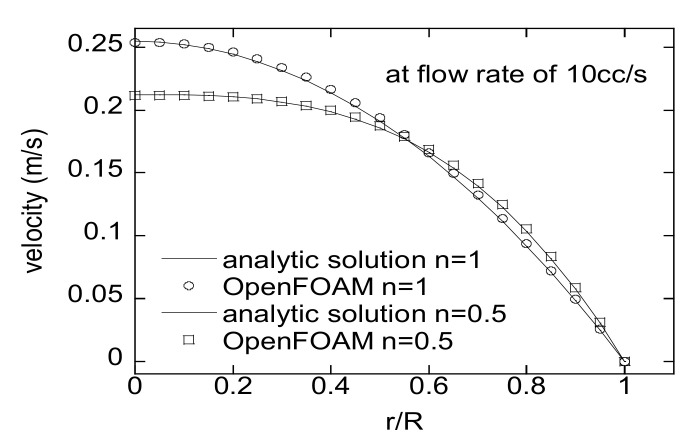
Comparison of an analytic solution and that by OpenFOAM for a Newtonian fluid and a virtual power-law fluid (PLF) of *n* = 0.5.

**Figure 8:**
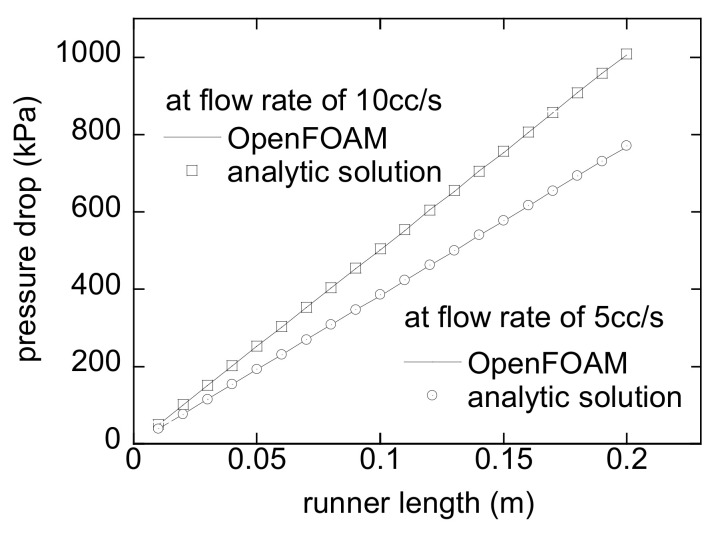
The pressure drop along the flow direction, OpenFOAM, and analytic solutions by Equation (13) for a PLF of *n* = 0.388, $K=1842.4\;\mathrm{Pa}\cdot s^{n}$ and *ρ* = 891.63 kg/m^3^.

In order to check the accuracy of OpenFOAM, the velocity profiles obtained by OpenFOAM and the analytic solution by Equation (15) have been compared for a virtual fluid with $\rho =1000\;{\mathrm{kg}/m}^{3}$ and $K=1000\;\mathrm{Pa}\cdot s^{n}$. A tube with 10 mm diameter and 100 mm length is considered here. Figure 7 shows the results for *n* = 1 and *n* = 5 at a flow rate of 10 cc/s. The numerical velocities exactly agree with the analytical results. Then, to check the validity of the developed method in a straight runner, the pressure drops by HRPDC are compared with those by Equation (16) along a 200 mm long runner with a diameter of 10 mm. In this case, a PLF for a polymer melt has been chosen to perform the verification under a more realistic condition. Refer to the caption of Figure 8 for the viscosity of the PLF. The results by both methods exactly match at flow rates of 5 cc/s and 10 cc/s as shown in Figure 8.

### 4.2. Implemented System

**Figure 9:**
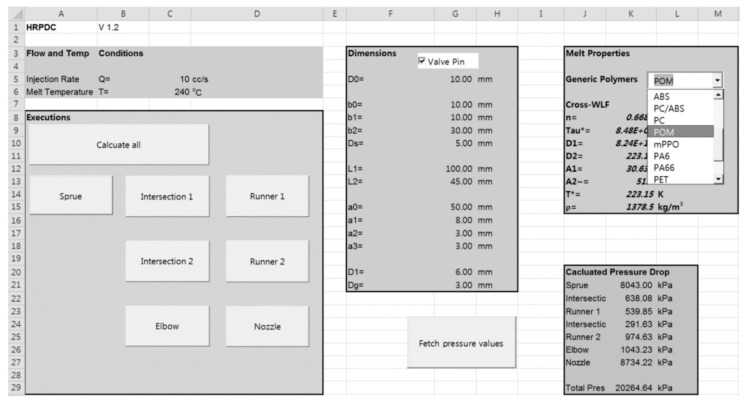
Hot runner pressure drop calculator (HRPDC) interface developed in an Excel sheet for execution, variable input, and material properties.

The interface of the HRPDC is implemented in an Excel sheet. The sheet presents the buttons for executions. In addition to buttons created for calculation of each subdomain, a button for the total pressure drop that performs a batch parallel calculation has been placed as shown in Figure 9. Upon completion of all the calculations, the results are collected through VBA and retrieved in the cells.

All the geometric design dimensions, shown in Figure 6, are put into the cells in the same sheet. These are the independent variables in HRPDC. With the input numbers here, the OpenFOAM utilities, blockMesh and transformPoints, will set the final mesh for HRPDC. Furthermore, in the sheet, the properties of the thermoplastic melts are input in the cells. For the straight runners without pin, a two-dimensional simulation is viable. However, OpenFOAM is inherently a three-dimensional CFD program. Thus, in this case, a wedge-type mesh has been built. In blockMeshDict, a thin sector from the circular cross-section is considered and the sides are specified as wedge. Where prebuilt meshes are required, the meshes have been built in Ansys and stored in the format of the Fluent mesh. Then, they are converted to OpenFOAM mesh by the utility, fluent3DMeshToFoam.

The size of mesh was determined based on repeated tests. Initially, a sufficiently dense meshes were created considering the cell Reynolds number for the highest flow rate case, 300 cc/s in the test cases. Then, the test was repeated, made sparser checking the accuracy and the stability of the solution. By doing so, the sparsest possible meshes have been set for the fast run. The prebuilt mesh for intersection 2 can be found in Figure 1. 

For some representative thermoplastics, the material can be selective in a dropdown box. On selection of a material in the dropdown box, the Cross-WLF coefficients of the corresponding material show up in the cells and they are ready for use in the calculations. Otherwise, one can directly input numbers in the cells. The programming with the Excel VBA follows the similar way conventional Windows Visual Basic codes are written, using the concept of properties and methods of each object.

### 4.3. Comparison with Simulation Software

**Figure 10:**
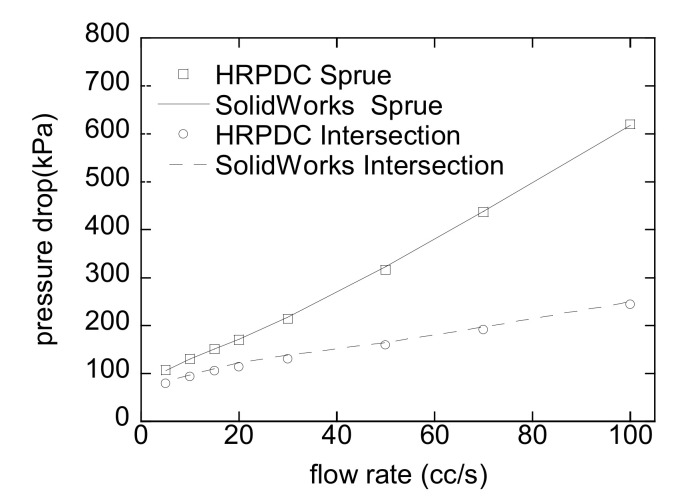
Comparison of the results by HRPDC and SolidWorks Flow Simulation. Refer to Figure 8 for the viscosity and Figure 9 for the dimensions.

To make it sure, the HRPDC accurately calculates the pressure drop during the steady isothermal flow, the results for two different sections have been compared with those by another commercial tool, SolidWorks Flow Simulation 2011. Figure 10 shows the compared pressure drop for the sprue and the intersection in Figure 10. The results by both tools agree well within the presented range of flow rate.

**Figure 11:**
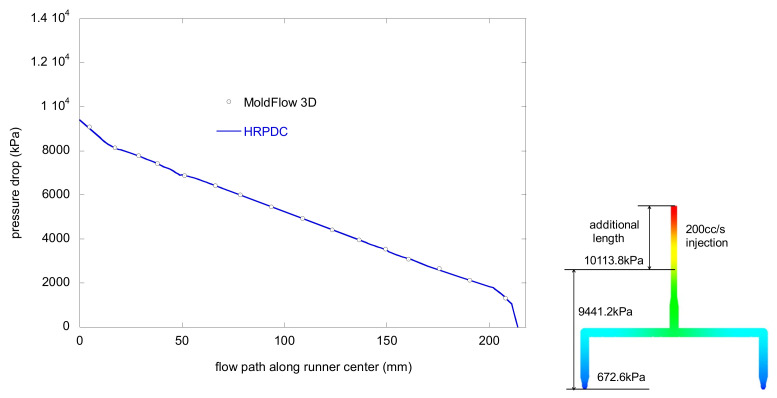
Pressure drop along the flow path for flow rate of 200 cc/s at 240 °C for the dimensions in Figure 9.

For a simple HRS with two nozzles, the pressure profiles by MoldFlow and HRPDC have been compared along the flow path for 200 cc/s in Figure 11. The tested material was PP at 240 °C whose viscosity is presented in Table 1. Moreover, refer to Figure 9 for the dimensions. On the right hand side of the figure, how the pressure drop is evaluated from a MoldFlow simulation is presented together conceptually. The sprue was extended by giving some additional length to make the inlet flow fully developed. However, the pressure values are not directly obtained by the examination tool in MoldFlow. The values are estimated by interpolation with the Patran export file from MoldFlow for accurate comparison. The details of the data processing method have been described in [2]. As mentioned previously, to overcome isothermal limitation of HRPDC, the results are calculated twice. First, the mean pressure of the section is calculated to evaluate the density. Then, the final result is obtained with the density. Another thing that should be mentioned is that the pressure profile for MoldFlow is acquired as soon as the gate is wet.

**Figure 12:**
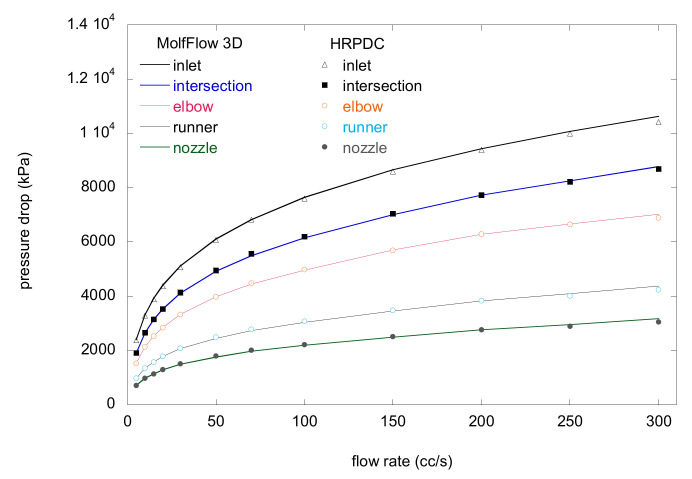
Comparison of the results by HRPDC and MoldFlow at 240 °C for the dimensions in Figure 9.

In Figure 12, the pressure drops have been compared for a wide range from 5 cc/s to 300 cc/s at the interconnecting points. For each interconnecting point, the compared pressure drops by the both methods coincide well. The errors become larger as the flow rate increases, resulting in the final error for the 300 cc/s case 1.97%. Errors are mostly observed in the sprue section as can be noticed in the figure. Although the density is corrected, it cannot compensate for all the possible errors.

### 4.4. Runner Diameter and Length

**Figure 13:**
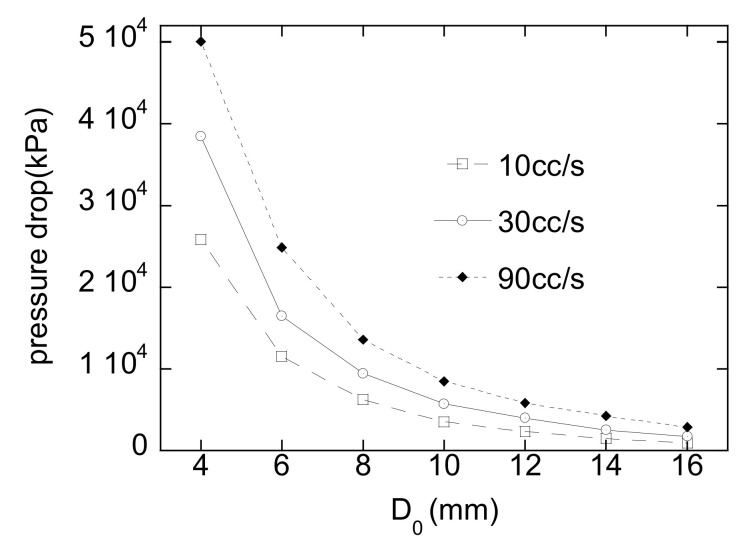
Given a *L*_1_, pressure drop in a four-nozzle HR along with *D*_0_ for different flow rates (10, 30, and 90 cc/s).

Consider that one has to determine the diameter in Figure 6b during a design process of a HR. For a fixed runner length, the pressure drop is calculated along with the runner diameter for different flow rates. Figure 13 shows the calculated pressure drop for the HRS with four nozzles shown in Figure 1. For an allowable maximum pressure drop and a flow rate, the minimum runner diameter can be determined from this result. For example, when the maximum pressure drop in the HR and the flow rate are 10^4^ kPa and 90 cc/s, respectively, the diameter *D*_0_ should be at least 8.6 mm. There could be some constraints other than rheological ones that need to be taken into account for determination of such a variable. The residence time of HR is an important factor to be regulated since the melts inside HR start to be degraded by chain scission and cross-linking eventually resulting in yellow or black specks. The diameter of an HRS should be as small as possible in this regard. On the other hand, the machinability of a runner hole with a gun drill limits the minimum diameter. Therefore, there are lower and upper bounds for the diameter.

Consider a design case where we have to determine *L*_1_ and *D*_0_ shown Figure 6b. Figure 12 shows the pressure drop calculated for different *L*_1_ and *D*_0_. When an allowable pressure drop is set, one can obtain the design windows for those variables. The HRPDC can calculate the pressure drops for different design variables in a batch process. Then, the results can be graphically presented or tabulated and then can be conveniently used for design of a HRS.

### 4.5. Computational Aspects and Limitations

The key of this work is rapid calculation. To do so, simpleFoam can be run in a parallel fashion using all the CPU cores. Even without the parallel setup in OpenFOAM, Excel can initiate the process for each subdomain in a separate thread. Thus, there is no problem in maximally exploiting the resources in a local machine. In the iteration of simpleFoam, the criterion for stoppage should be set as big as possible not to continue unnecessary computation. The criterion has been set to obtain a solution that has an error of 0.1% in the case for Figure 8. Moreover, the CPU time for the MoldFlow model with 145,611 elements including the cavity was 149.52 s with Intel Core i9-9900 K 3.6 GHz CPU. The HRPDC has run physically for 32.1 s by allocating each subsection task to separate core. Note that HRPDC ran twice for the aforementioned density correction.

Apart from the computational time, the time required for preprocessing can be dramatically saved with HRPDC. This benefit comes from the predesigned constraints of the HRS. If the design criteria are changed, HRPDC should be updated accordingly to accommodate it. The isothermal condition can limit its application in some unusual cases where the manifold and nozzle are under different temperature. In this case, the viscosity model should be differently imposed. As has been mentioned earlier, the density change due to pressure can induce large.

## 5. Conclusions

This work has presented a method for rapid calculation of pressure drip in hot runners. Assuming steady-state and isothermality, a steady Navier–Stokes equation has been solved to obtain the pressure drop in the subdomains, which are defined by dividing the whole domain along the flow path. A computer method that can estimate pressure drops according to the hot runner dimensions are established. We have verified that the pressure drop can be accurately estimated by adding up those calculated in all the subdomains based on comparisons with MoldFlow. The computer utility has been built using two existing computer tools, Excel and OpenFOAM. The communication structure between these two tools has been implemented by the Excel VBA. It has also been shown that the developed tool, the HRPDC, can be employed for practical design of hot runner dimensions.

Although the model has a couple of assumptions, the proposed method is not a rough tool with big approximations. The method has utilized the standardized characteristics of hot runner manifolds and runners in contrast to those of mold cavities. For a given hot runner product line, the geometric models can be reused for different orders. The developed tool has been adopted for real-world design processes of hot runner systems.

## Figures and Tables

**Table 1:** Coefficients of Cross-WLF model for a generic PP [29].

*n*	0.2751
*τ* *	24200 Pa
*D_1_*	4.66 × 10^12^ Pa·s
*D_2_*	263.15 K
*A_1_*	26.12
*A_2_*	51.6 K

**Table 2:** Constants of the *p*-*v*-*T* equation for PP [29].

*C*	0.0894
*b*_5_	443.15 K
*b*_6_	1.12 × 10^−7^ K/Pa
*b*_1m_	0.001304 m^3^/kg
*b*_2m_	1.037 × 10^−6^ m^3^/kgK
*b*_3m_	8.48518 × 10^7^ Pa
*b*_4m_	0.00635 K^−1^

**Table 3:** Subdomains.

Subdomain	Mesh	Calculation Method	Subdomain Dimension	Design Variables
sprue	axisymmetric	FVM analytical	*b*_2a_ = *b*_2_ − 2*D*_0_	*b*_0_, *b*_1_, *b*_2_
intersection 1	three-dimensional	FVM	inlet to center: 2*D*_0_ center to outlet: 2*D*_0_	none (dependent)
runner 1	axisymmetric	FVM analytical	*L*_1a_ = *L*_1_ − 4*D*_0_	*L*_1_
intersection 2	three-dimensional	FVM	inlet to center: 2*D*_0_ center to outlet: 2*D*_0_	none (dependent)
runner 2	axisymmetric	FVM analytical	*L*_2a_ = *L*_2_ − 3*D*_0_	*L*_2_
elbow	three-dimensional	FVM	inlet to elbow: *D*_0_ elbow to outlet: 2*D*_0_	none (dependent)
nozzle without valve	axisymmetric	FVM analytical	*a*_0a_ = *a*_0_ − 2*D*_0_	*a*_0_, *a*_1_, *a*_2_, *a*_3_, *D*_1_, *D*_g_
nozzle with valve	three-dimensional	FVM analytical	fixed	*a*_0_, *a*_1_, *a*_2_, *a*_3_, *D*_1_, *D*_g_
